# ﻿New species of *Diaporthe* (Diaporthaceae, Diaporthales) from *Bauhiniavariegata* in China

**DOI:** 10.3897/mycokeys.108.128983

**Published:** 2024-09-13

**Authors:** Yaquan Zhu, Lei Ma, Han Xue, Yong Li, Ning Jiang

**Affiliations:** 1 Key Laboratory of Biodiversity Conservation of National Forestry and Grassland Administration, Ecology and Nature Conservation Institute, Chinese Academy of Forestry, Beijing 100091, China Ecology and Nature Conservation Institute, Chinese Academy of Forestry Beijing China; 2 Forest Pest Control and Quarantine Station of Tonghua County, Tonghua 134001, China Forest Pest Control and Quarantine Station of Tonghua County Tonghua China

**Keywords:** Diaporthales, morphology, multi-gene phylogeny, taxonomy, two new taxa

## Abstract

*Diaporthe* species are known as endophytes, saprobes and pathogens infecting a wide range of plants and resulting in important crop diseases. In the present study, four strains of *Diaporthe* were obtained from diseased leaves of *Bauhiniavariegata* in Guangdong Province, China. Phylogenetic analyses were conducted to identify these strains using five gene regions: internal transcribed spacer (ITS), calmodulin (*cal*), histone H3 (*his3*), translation elongation factor 1-α (*tef1*) and β-tubulin (*tub2*). The results combined with morphology revealed two new species of *Diaporthe* named *D.bauhiniicola* in *D.arecae* species complex and *D.guangzhouensis* in *D.sojae* species complex.

## ﻿Introduction

*Diaporthe* (syn. *Phomopsis*) is the type genus of Diaporthaceae in Diaporthales (Hyde et al. 2014; Maharachchikumbura et al. 2016). Before the implementation of “one fungus, one name”, it has been a common practice to use two names for the fungal species with pleomorphic life cycles (Taylor 2011). The genus *Diaporthe* established in 1870 predates *Phomopsis* established in 1905, thus *Diaporthe* is recommended for use (Rossman et al. 2015). More than 1200 epithets for *Diaporthe* have been listed in Index Fungorum with names often based on host association (http://www.indexfungorum.org/, accessed June 2024).

The teleomorph of *Diaporthe* is characterized by aggregated spherical ascomata with tapering necks, unitunicate, 8-spored, elongate to clavate asci, and septate or aseptate, elongated to elliptical, hyaline ascospores with larger guttules at center and smaller ones at the ends (Senanayake et al. 2018; Yang et al. 2020). The anamorph is characterized by black, ostiolate pycnidia containing cylindrical phialides often producing three types of hyaline, aseptate conidia called α-conidia, β-conidia and γ-conidia (Udayanga et al. 2012a; Dissanayake et al. 2017; Fan et al. 2018). The α-conidia and β-conidia are produced frequently, but the γ-conidia are rarely observed (Gomes et al. 2013).

*Diaporthe* species are associated with a wide range of plant hosts as pathogens, endophytes and saprobes of crops, forest trees and ornamentals (Farr et al. 2002a; Crous 2005; Udayanga et al. 2012b, 2014a, 2014b, 2015; Jiang et al. 2021; Zhu et al. 2023). As plant pathogens, *Diaporthe* species cause severe diseases, e.g., leaf spots, blights, dieback, scab, decay, stem end rots and wilt of many economically important plants including species of *Citrus* (Guarnaccia and Crous 2017), *Macadamia* (Wrona et al. 2020), *Rosa* (Caio et al. 2021), *Vaccinium* (Farr et al. 2002b), *Vitis* (Manawasinghe et al. 2019) and many more (Yang et al. 2018, 2021; Guarnaccia et al. 2020; Guo et al. 2020; Ariyawansa et al. 2021). In addition, *Diaporthe* species can live inside the healthy host tissues as endophytes (Huang et al. 2015; Dong et al. 2021). In addition, species of *Diaporthe* have been also reported as saprobes from different woody hosts (Dissanayake et al. 2020).

Species identification of *Diaporthe* has traditionally been based on host as well as morphological characters such as the size and shape of fruiting bodies and spores (Mostert et al. 2001; Santos and Phillips 2009). However, recent studies have shown that many species of *Diaporthe* are not host-specific i.e., one species may infect more than one host species (Vrandecic et al. 2011; Bai et al. 2015; Zhang et al. 2018; Huang et al. 2021; Sun et al. 2021; Cao et al. 2022). Moreover, many *Diaporthe* species that are morphologically similar have proven to be genetically distinct (van Rensburg et al. 2006; Yang et al. 2018). Phylogenetic analysis using a five-locus dataset (ITS-*tef1*-*tub2*-*cal*-*his3*) has been widely used to identify species of *Diaporthe* species (Santos et al. 2017; Marin-Felix et al. 2019; Hilário et al. 2021b; Norphanphoun et al. 2022). *Diaporthe* was clustered into 13 groups, namely *D.arecae*, *D.biconispora*, *D.carpini*, *D.decedens*, *D.eres*, *D.oncostoma*, *D.pustulata*, *D.rudis*, *D.scobina*, *D.sojae*, *D.toxica*, *D.varians* and *D.vawdreyi* species complexes and nine singletons as *D.acerina*, *D.acutispora*, *D.crataegi*, *D.multiguttulata*, *D.ocoteae*, *D.perjuncta*, *D.pseudoalnea*, *D.spartinicola* and *D.undulata* based on multilocus phylogeny (Norphanphoun et al. 2022; Hongsanan et al. 2023).

*Bauhiniavariegata* is a flowering plant species belonging to Fabaceae. It is native to China and cultivated as an ornamental tree in subtropical and tropical climate for its scented flowers. The aim of the present study was to identify new isolates collected from diseased leaves of *Bauhiniavariegata* in China following the combined approaches of morphology and phylogeny in the genus *Diaporthe*.

## ﻿Materials and methods

### ﻿Isolation and morphological characterization

In 2022, a plant disease investigation was conducted in Guangdong Province, China. Small and irregular leaf spots were observed on the leaves of *Bauhiniavariegata*, and 14 leaves were collected for isolation. The leaves were firstly surface-sterilized for 1 min in 75% ethanol, 3 min in 1.25% sodium hypochlorite and 1 min in 75% ethanol, rinsed for 2 min in distilled water and blotted on dry sterile filter paper. Then, the discolored areas were cut into 0.5 × 0.5 cm pieces and transferred to the surface of potato dextrose agar plates (PDA; 200 g potatoes, 20 g dextrose, 20 g agar per litre), incubated at 25 °C to obtain pure cultures. The cultures were deposited in the China Forestry Culture Collection Center (CFCC; http://cfcc.caf.ac.cn/) and the specimen was deposited in the Herbarium of the Chinese Academy of Forestry (CAF; http://museum.caf.ac.cn/).

The isolates were grown on PDA, MEA and SNA plates, incubated at 25 °C under a 12 h near-ultraviolet light/12 h dark cycle to induce sporulation. Colony characters and pigment production on PDA, MEA and SNA were noted for the 10-day culture. Microscopic structures of the fungi growing on medium were mounted in water and examined under an Axio Imager 2 microscope (Zeiss, Oberkochen, Germany). At least 30 measurements were made for each structure examined.

### ﻿DNA extraction, amplification and sequencing

The genomic DNA was extracted from the fresh mycelium harvested from PDA plates after seven days using a cetyltrimethylammonium bromide (CTAB) method (Doyle and Doyle 1990). For initial genus confirmation, the internal transcribed spacer (ITS) region was sequenced. After confirmation of *Diaporthe* species, four additional gene regions coding for translation elongation factor 1-alpha (*tef1*), beta-tubulin (*tub2*), calmodulin (*cal*) and his-tone H3 (*his3*) were sequenced. The primer pairs and amplification conditions for each of the above-mentioned gene regions are provided in Table 1.

**Table 1.** Loci assessed in this study with used PCR primers and program.

Loci	Primers	PCR: Thermal Cycles: (Annealing Temp. in Bold)	Reference
ITS	ITS1f/ITS4	(95 °C: 30 s, **48 °C**: 30 s, 72 °C: 1 min) × 35 cycles	White et al. 1990
*cal*	CAL228F/CAL737R	(95 °C: 15 s, **54 °C**: 20 s, 72 °C: 1 min) × 35 cycles	Carbone and Kohn 1999
*his3*	CYLH3F/H3-1b	(95 °C: 30 s, **57 °C**: 30 s, 72 °C: 1 min) × 35 cycles	Crous et al. 2004; Glass and Donaldson 1995
*tef1*	EF1-728F/EF1-986R	(95 °C: 15 s, **54 °C**: 20 s, 72 °C: 1 min) × 35 cycles	Carbone and Kohn 1999
*tub2*	T1(Bt2a)/Bt2b	(95 °C: 30 s, **55 °C**: 30 s, 72 °C: 1 min) × 35 cycles	Glass and Donaldson 1995; O’Donnell and Cigelnik 1997

A PCR reaction was conducted in a 20 µL reaction volume, and the components were as follows: 1 µL DNA template (20 ng/μL), 1 µL forward 10 µM primer, 1 µL reverse 10 µM primer, 10 µL T5 Super PCR Mix (containing Taq polymerase, dNTP and Mg2+, Beijing Tisingke Biotech Co., Ltd., Beijing, China), and 7 µL sterile water. Amplifications were performed using a T100 Thermal Cycler (Bio-Rad, Hercules, CA, USA). All amplified PCR products were evaluated visually with 1.4% agarose gels stained with ethidium bromide and PCR positive products sent to Sangon Biotech (Shanghai) Co., Ltd., (Beijing, China) for sequencing. Strands were sequenced in both directions using PCR primers. The new sequences generated in this study, as well as the reference sequences of all isolates used in the present study, are listed in Table 2.

**Table 2.** Isolates and GenBank accession numbers used in the phylogenetic analyses of *Diaporthe*.

Species	Location	Host	Strain	GenBank Accession Number				
				ITS	*tef1*	*tub2*	*cal*	*his3*
*Diaportheabsenteum*	China	*Camelliasinensis*	LC3429*	KP267897	KP267971	KP293477	NA	KP293547
*D.absenteum*	China	*Camelliasinensis*	LC3564	KP267912	KP267986	KP293492	NA	KP293559
*D.acaciarum*	Tanzania	*Acaciatortilis*	CBS 138862*	KP004460	NA	KP004509	NA	KP004504
*D.acericola*	Italy	*Acernegundo*	MFLUCC 17-0956*	KY964224	KY964180	KY964074	KY964137	NA
*D.aceris*	Japan	*Acer* sp.	LC8112	KY491547	KY491557	KY491567	KY491575	NA
*D.actinidiae*	New Zealand	*Actinidiadeliciosa*	ICMP 13683*	KC145886	KC145941	NA	NA	NA
*D.acuta*	China	*Pyruspyrifolia*	CGMCC 3.19600*	MK626957	MK654802	MK691225	MK691124	MK726161
*D.alangii*	China	*Alangiumkurzii*	CFCC 52556*	MH121491	MH121533	MH121573	MH121415	MH121451
*D.alangii*	China	*Alangiumkurzii*	CFCC 52557	MH121492	MH121534	MH121574	MH121416	MH121452
*D.alnea*	Netherlands	*Alnus* sp.	CBS 146.46	KC343008	KC343734	KC343976	KC343250	KC343492
*D.amaranthophila*	Japan	*Amaranthustricolor*	MAFF 246900	LC459575	LC459577	LC459579	LC459583	LC459581
*D.ambigua*	South Africa	*Pyruscommunis*	CBS 114015*	KC343010	KC343736	KC343978	KC343252	KC343494
*D.angelicae*	Austria	*Heracleumsphondylium*	CBS 111592*	KC343027	KC343753	KC343995	KC343269	KC343511
*D.anhuiensis*	China	*Cunninghamialanceolata*	CNUCC 201901*	MN219718	MN224668	MN227008	MN224549	MN224556
*D.arctii*	Austria	*Arctiumlappa*	CBS 139280*	KJ590736	KJ590776	KJ610891	KJ612133	KJ659218
*D.arecae*	India	*Arecacatechu*	CBS 161.64*	KC343032	KC343758	KC344000	KC343274	KC343516
*D.arengae*	Hong Kong	*Arengaengleri*	CBS 114979*	KC343034	KC343760	KC344002	KC343276	KC343518
*D.arezzoensis*	Italy	*Cytisus* sp.	MFLUCC 15-0127	MT185503	NA	NA	NA	NA
*D.aseana*	Thailand	Unidentified dead leaf	MFLUCC 12-0299a*	KT459414	KT459448	KT459432	KT459464	NA
*D.australiana*	Australia	*Macadamia*	CBS 146457	MN708222	MN696522	MN696530	NA	NA
***D.bauhiniicola***	**China**	***Bauhiniavariegata***	CFCC 58154*	**PP864723**	**PP938599**	**PP938603**	**PP938607**	**PP938611**
***D.bauhiniicola***	**China**	***Bauhiniavariegata***	**GZ13B**	**PP864724**	**PP938600**	**PP938604**	**PP938608**	**PP938612**
*D.batatas*	USA	*Ipomoeabatatas*	CBS 122.21*	KC343040	KC343766	KC344008	KC343282	KC343524
*D.beilharziae*	Australia	*Indigoferaaustralis*	BRIP 54792*	JX862529	JX862535	KF170921	NA	NA
*D.biconispora*	China	*Citrusgrandis*	ZJUD62	KJ490597	KJ490476	KJ490418	MT227578	KJ490539
*D.biguttulata*	China	*Citruslimon*	ZJUD47*	KJ490582	KJ490461	KJ490403	NA	KJ490524
*D.brasiliensis*	Brazil	*Aspidosperma* sp.	CBS 133183*	KC343042	KC343768	KC344010	KC343284	KC343526
*D.caatingaensis*	Brazil	*Tacingainamoena*	CBS 141542*	KY085927	KY115603	KY115600	NA	KY115605
*D.camelliae-oleiferae*	China	*Camelliaoleifera*	HNZZ027*	MZ509555	MZ504707	MZ504718	MZ504685	MZ504696
*D.caryae*	China	*Caryaillinoensis*	CFCC 52563*	MH121498	MH121540	MH121580	MH121422	MH121458
*D.caryae*	China	*Caryaillinoensis*	CFCC 52564	MH121499	MH121541	MH121581	MH121423	MH121459
*D.cercidis*	China	*Cercischinensis*	CFCC 52565*	MH121500	MH121542	MH121582	MH121424	MH121460
*D.cercidis*	China	*Cercischinensis*	CFCC 52566	MH121501	MH121543	MH121583	MH121425	MH121461
*D.chiangraiensis*	Thailand	*Bauhinia* sp.	MFLUCC 17-1669*	MF190119	MF377598	NA	NA	NA
*D.chrysalidocarpi*	China	*Chrysalidocarpuslutescens*	SAUCC194.35	MT822563	MT855760	MT855876	MT855646	MT855532
*D.cichorii*	Italy	*Cichoriumintybus*	MFLUCC 17-1023*	KY964220	KY964176	KY964104	KY964133	NA
*D.cinmomi*	China	*Cinnamomum* sp.	CFCC 52569*	MH121504	MH121546	MH121586	NA	MH121464
*D.cinmomi*	China	*Cinnamomum* sp.	CFCC 52570	MH121505	MH121547	MH121587	NA	MH121465
*D.citriasiana*	China	*Citrusunshiu*	CGMCC 3.15224*	JQ954645	JQ954663	KC357459	KC357491	KJ490515
*D.columnaris*	USA	*Vacciniumvitisidaea*	AR3612*	AF439625	NA	NA	NA	NA
*D.compacta*	China	*Camelliasinensis*	CGMCC 3.17536*	KP267854	KP267928	KP293434	NA	KP293508
*D.convolvuli*	Turkey	*Convolvulusarvensis*	CBS 124654*	KC343054	KC343780	KC344022	KC343296	KC343538
*D.cucurbitae*	Canada	*Cucumis* sp.	DAOM 42078*	KM453210	KM453211	KP118848	NA	KM453212
*D.cuppatea*	South Africa	*Aspalathuslinearis*	CBS 117499*	KC343057	KC343783	KC344025	KC343299	KC343541
*D.cyatheae*	Taiwan	*Cyathealepifera*	YMJ 1364*	JX570889	KC465406	KC465403	KC465410	NA
*D.discoidispora*	China	*Citrusunshiu*	ZJUD89*	KJ490624	KJ490503	KJ490445	NA	KJ490566
*D.drenthii*	Australia	*Macadamia*	CBS 146453	MN708229	MN696526	MN696537	NA	NA
*D.durionigena*	Vietnam	*Duriozibethinus*	VTCC 930005	MN453530	MT276157	MT276159	NA	NA
*D.endocitricola*	China	*Citrusmaxima*	ZHKUCC20-0012*	MT355682	MT409336	MT409290	MT409312	NA
*D.endophytica*	Brazil	*Schinusterebinthifolius*	CBS 133811*	KC343065	KC343791	KC344033	KC343307	KC343549
*D.eucalyptorum*	China	*Eucalyptus*	CBS 132525*	MH305525	NA	NA	NA	NA
*D.eugeniae*	Indonesia	*Eugeniaaromatica*	CBS 444.82*	KC343098	KC343824	KC344066	KC343340	KC343582
*D.fraxini-angustifoliae*	Australia	*Fraxinusangustifolia*	BRIP 54781*	JX862528	JX862534	KF170920	NA	NA
*D.fructicola*	Japan	*Passifloraedulis × P. edulis*	MAFF 246408*	LC342734	LC342735	LC342736	LC342738	LC342737
*D.fulvicolor*	China	*Pyruspyrifolia*	CGMCC 3.19601*	MK626859	MK654806	MK691236	MK691132	MK726163
*D.ganjae*	USA	*Cannabissativa*	CBS 180.91*	KC343112	KC343838	KC344080	KC343354	KC343596
*D.goulteri*	Australia	*Helianthusannuus*	BRIP 55657a*	KJ197290	KJ197252	KJ197270	NA	NA
*D.guangdongensis*	China	*Citrusmaxima*	ZHKUCC20-0014*	MT355684	MT409338	MT409292	MT409314	NA
*D.guangxiensis*	China	*Vitisvinifera*	JZB320094*	MK335772	MK523566	MK500168	MK736727	NA
***D.guangzhouensis***	**China**	***Bauhiniavariegata***a	CFCC 58151*	**PP864725**	**PP938601**	**PP938605**	**PP938609**	**PP938613**
***D.guangzhouensis***	**China**	***Bauhiniavariegata***	**GZ13E**	**PP864726**	**PP938602**	**PP938606**	**PP938610**	**PP938614**
*D.gulyae*	Australia	*Helianthusannuus*	BRIP 54025*	JF431299	JN645803	KJ197271	NA	NA
*D.guttulata*	China	Unknown	CGMCC 3.20100	MT385950	MT424685	MT424705	MW022470	MW022491
*D.helianthi*	Serbia	*Helianthusannuus*	CBS 592.81*	KC343115	KC343841	KC344083	KC343357	KC343599
*D.heterostemmatis*	China	*Heterostemmagrandiflorum*	SAUCC194.85*	MT822613	MT855925	MT855810	MT855692	MT855581
*D.hongkongensis*	China	*Dichroafebrífuga*	CBS 115448*	KC343119	KC343845	KC344087	KC343361	KC343603
*D.hordei*	Norway	*Hordeumvulgare*	CBS 481.92*	KC343120	KC343846	KC344088	KC343362	KC343604
*D.huangshanensis*	China	*Camelliaoleifera*	CNUCC 201903*	MN219729	MN224670	MN227010	NA	MN224558
*D.hubeiensis*	China	*Vitisvinifera*	JZB320123	MK335809	MK523570	MK500148	MK500235	NA
*D.hunanensis*	China	*Camelliaoleifera*	HNZZ023*	MZ509550	MZ504702	MZ504713	MZ504680	MZ504691
*D.infecunda*	Brazil	*Schinus* sp.	CBS 133812*	KC343126	KC343852	KC344094	KC343368	KC343610
*D.infertilis*	Suriname	*Camelliasinensis*	CBS 230.52*	KC343052	KC343778	KC344020	KC343294	KC343536
*D.kochmanii*	Australia	*Helianthusannuus*	BRIP 54033*	JF431295	JN645809	NA	NA	NA
*D.kongii*	Australia	*Portulacagrandifla*	BRIP 54031*	JF431301	JN645797	KJ197272	NA	NA
*D.krabiensis*	Thailand	marine based habitats	MFLUCC 17-2481*	MN047101	MN433215	MN431495	NA	NA
*D.leucospermi*	Australia	*Leucospermum* sp.	CBS 111980*	JN712460	KY435632	KY435673	KY435663	KY435653
*D.limonicola*	Malta	*Citruslimon*	CPC 28200*	NR_154980	MF418501	MF418582	MF418256	MF418342
*D.litchiicola*	Australia	*Litchichinensis*	BRIP 54900*	JX862533	JX862539	KF170925	NA	NA
*D.lithocarpi*	China	*Lithocarpusglabra*	CGMCC 3.15175*	KC153104	KC153095	KF576311	KF576235	NA
*D.longicolla*	USA	*Glycinemax*	FAU599*	KJ590728	KJ590767	KJ610883	KJ612124	KJ659188
*D.longispora*	Canada	*Ribes* sp.	CBS 194.36*	KC343135	KC343861	KC344103	KC343377	KC343619
*D.lusitanicae*	Portugal	*Foeniculumvulgare*	CBS 123212	KC343136	KC343862	KC344104	KC343378	KC343620
*D.lusitanicae*	Portugal	*Foeniculumvulgare*	CBS 123213*	MH863280	KC343863	KC344105	KC343379	KC343621
*D.malorum*	Portugal	*Malusdomestica*	CAA 734*	KY435638	KY435627	KY435668	KY435658	KY435648
*D.manihotia*	Rwanda	*Manihotutilissima*	CBS 505.76	KC343138	KC343864	KC344106	KC343380	KC343622
*D.masirevicii*	Australia	*Helianthusannuus*	BRIP 57892a*	KJ197276	KJ197239	KJ197257	NA	NA
*D.mayteni*	Brazil	*Maytenusilicifolia*	CBS 133185	KC343139	KC343865	KC344107	KC343381	KC343623
*D.megalospora*	Not stated	*Sambucuscanadensis*	CBS 143.27	KC343140	KC343866	KC344108	KC343382	KC343624
*D.melitensis*	Malta	*Citruslimon*	CPC 27873*	MF418424	MF418503	MF418584	MF418258	MF418344
*D.melonis*	USA	*Cucumismelo*	CBS 507.78*	KC343142	KC343868	KC344110	KC343384	KC343626
*D.melonis*	Indonesia	*Glycinesoja*	CBS 435.87	KC343141	KC343867	KC344109	KC343383	KC343625
*D.middletonii*	Australia	*Rapistrumrugostrum*	BRIP 54884e*	KJ197286	KJ197248	KJ197266	NA	NA
*D.millettiae*	China	*Millettiareticulata*	GUCC9167*	MK398674	MK480609	MK502089	MK502086	NA
*D.minusculata*	China	saprobic on decaying wood	CGMCC 3.20098*	MT385957	MT424692	MT424712	MW022475	MW022499
*D.miriciae*	Australia	*Helianthusannuus*	BRIP 54736j*	KJ197282	KJ197244	KJ197262	NA	NA
*D.musigena*	Australia	*Musa* sp.	CBS 129519*	KC343143	KC343869	KC344111	KC343385	KC343267
*D.myracrodruonis*	Brazil	*Astroniumurundeuva*	URM 7972*	MK205289	MK213408	MK205291	MK205290	17
*D.nelumbonis*	Taiwan	*Nelumbonucifera*	R. Kirschner 4114*	KT821501	NA	LC086652	NA	NA
*D.neoarctii*	USA	*Ambrosiatrifi*	CBS 109490*	KC343145	KC343871	KC344113	KC343387	KC343629
*D.neoraonikayaporum*	Thailand	*Tectonagrandis*	MFLUCC 14-1136*	KU712449	KU749369	KU743988	KU749356	NA
*D.oculi*	Japan	*Homosapiens*	HHUF 30565*	LC373514	LC373516	LC373518	NA	NA
*D.osmanthi*	China	*Osmanthusfragrans*	GUCC9165*	MK398675	MK480610	MK502091	MK502087	NA
*D.ovalispora*	China	*Citruslimon*	CGMCC 3.17256*	KJ490628	KJ490507	KJ490449	NA	KJ490570
*D.oxe*	Brazil	*Maytenusilicifolia*	CBS 133186*	KC343164	KC343890	KC344132	KC343406	KC343648
*D.pandanicola*	Thailand	*Pandanus* sp.	MFLUCC 17-0607*	MG646974	NA	MG646930	NA	NA
*D.paranensis*	Brazil	*Maytenusilicifolia*	CBS 133184*	KC343171	KC343897	KC344139	KC343413	KC343655
*D.pascoei*	Australia	*Perseaamericana*	BRIP 54847*	JX862532	JX862538	KF170924	NA	NA
*D.passiflorae*	South America	*Passiflaedulis*	CBS 132527*	JX069860	KY435633	KY435674	KY435664	KY435654
*D.passifloricola*	Malaysia	*Passiflorafoetida*	CBS 141329*	KX228292	NA	KX228387	NA	KX228367
*D.perseae*	Netherlands	*Perseagratissima*	CBS 151.73*	KC343173	KC343899	KC343141	KC343415	KC343657
*D.pescicola*	China	*Prunuspersica*	MFLUCC 16-0105*	KU557555	KU557623	KU557579	KU557603	NA
*D.phaseolorum*	USA	*Phaseolusvulgaris*	AR4203*	KJ590738	KJ590739	KJ610893	KJ612135	KJ659220
*D.phoenicicola*	India	*Arecacatechu*	CBS 161.64*	MH858400	GQ250349	JX275440	JX197432	NA
*D.podocarpi-macrophylli*	China	*Podocarpusmacrophyllus*	CGMCC 3.18281*	KX986774	KX999167	KX999207	KX999278	KX999246
*D.pseudobauhiniae*	Thailand	*Bauhinia* sp.	MFLU 17-1670	MF190118	MF377599	NA	NA	NA
*D.pseudobauhiniae*	Thailand	*Bauhinia* sp.	MFLUCC 17-1669*	MF190119	MF377598	NA	NA	NA
*D.pseudolongicolla*	Serbia	*Glycinemax*	PL42*	JQ697843	JQ697856	NA	NA	NA
*D.pseudolongicolla*	Croatia	*Glycinemax*	CBS 127269	KC343155	KC343881	KC344123	KC343397	KC343639
*D.pseudomangiferae*	Dominican Republic	*Mangiferaindica*	CBS 101339*	KC343181	KC343907	KC344149	KC343423	KC343665
*D.pseudooculi*	Japan	*Homosapiens*	HHUF 30617*	NR_161019	LC373517	LC373519	NA	NA
*D.pseudophoenicicola*	Spain	*Phoenixdactylifera*	CBS 462.69*	KC343184	KC343910	KC344152	KC343426	KC343668
*D.pseudophoenicicola*	Iraq	*Mangiferaindica*	CBS 176.77	KC343183	KC343909	KC344151	KC343425	KC343667
*D.pterocarpicola*	Thailand	*Pterocarpusindicus*	MFLUCC 10-0580a*	JQ619887	JX275403	JX275441	JX197433	NA
*D.pyracanthae*	Portugal	*Pyracanthacoccinea*	CBS 142384*	KY435635	KY435625	KY435666	KY435656	KY435646
*D.racemosae*	South Africa	*Euclearacemosa*	CPC 26646*	MG600223	MG600225	MG600227	MG600219	MG600221
*D.raonikayaporum*	Brazil	*Spondiasmombin*	CBS 133182*	KC343188	KC343914	KC344156	KC343430	KC343672
*D.rhodomyrti*	China	*Rhodomyrtustomentosa*	CFCC 53101	MK432643	MK578119	MK578046	MK442965	MK442990
*D.rhodomyrti*	China	*Rhodomyrtustomentosa*	CFCC 53102	MK432644	MK578120	MK578047	MK442966	MK442991
*D.rosae*	Thailand	*Rosa* sp.	MFLUCC 17-2658*	MG828894	NA	MG843878	MG829273	NA
*D.rosiphthora*	Brazil	*Rosa* sp.	COAD 2914*	MT311197	MT313693	NA	MT313691	NA
*D.rossmaniae*	Portugal	*Vacciniumcorymbosum*	CAA762*	MK792290	MK828063	MK837914	MK883822	MK871432
*D.sackstonii*	Australia	*Helianthusannuus*	BRIP 54669b*	KJ197287	KJ197249	KJ197267	NA	NA
*D.salinicola*	Thailand	*Xylocarpus* sp.	MFLU 18-0553*	MN047098	MN077073	NA	NA	NA
*D.sambucusii*	China	*Sambucuswilliamsii*	CFCC 51986*	KY852495	KY852507	KY852511	KY852499	KY852503
*D.sambucusii*	China	*Sambucuswilliamsii*	CFCC 51987	KY852496	KY852508	KY852512	KY852500	KY852504
*D.schimae*	China	*Schimasuperba*	CFCC 53103*	MK432640	MK578116	MK578043	MK442962	MK442987
*D.schimae*	China	*Schimasuperba*	CFCC 53104	MK432641	MK578117	MK578044	MK442963	MK442988
*D.schini*	Brazil	*Schinusterebinthifolius*	CBS 133181*	KC343191	KC343917	KC344159	KC343433	KC343675
*D.schoeni*	Italy	*Schoenusnigricans*	MFLU 15-1279*	KY964226	KY964182	KY964109	KY964139	
*D.sclerotioides*	Netherlands	*Cucumissativus*	CBS 296.67*	KC343193	KC343919	KC344161	KC343435	KC343677
*D.searlei*	Australia	*Macadamia*	CBS 146456*	MN708231	NA	MN696540	NA	NA
*D.sennae*	China	*Sennabicapsularis*	CFCC 51636*	KY203724	KY228885	KY228891	KY228875	NA
*D.sennae*	China	*Sennabicapsularis*	CFCC 51637	KY203725	KY228886	KY228892	KY228876	NA
*D.serafiniae*	Australia	*Helianthusannuus*	BRIP 55665a*	KJ197274	KJ197236	KJ197254	NA	NA
*D.siamensis*	Thailand	*Dasymaschalon* sp.	MFLUCC 10-0573a*	JQ619879	JX275393	JX275429	JX197423	NA
*D.sinensis*	China	*Amaranthus* sp.	ZJUP0033-4*	MK637451	MK660449	MK660447	NA	MK660451
*D.sojae*	USA	*Glycinemax*	FAU635*	KJ590719	KJ590762	KJ610875	KJ612116	KJ659208
*D.spinosa*	China	*Pyruspyrifolia*	CGMCC 3.19602*	MK626849	MK654811	MK691234	MK691129	MK726156
*D.stewartii*	Not stated	*Cosmosbipinnatus*	CBS 193.36*	MH867279	GQ250324	JX275421	JX197415	NA
*D.subellipicola*	China	On dead wood	KUMCC 17-0153*	MG746632	MG746633	MG746634	NA	NA
*D.subordinaria*	New Zealand	*Plantagolanceolata*	CBS 464.90*	KC343214	KC343940	KC344182	KC343456	KC343698
*D.taiwanensis*	Taiwan	*Ixorachinensis*	NTUCC 18-105-1*	MT241257	MT251199	MT251202	MT251196	NA
*D.taoicola*	China	*Prunuspersica*	MFLUCC 16-0117*	KU557567	KU557635	KU557591	NA	NA
*D.tarchonanthi*	South Africa	*Tarchonanthuslittoralis*	CBS 146073*	MT223794	NA	MT223733	NA	MT223759
*D.tecomae*	Brazil	*Tabebuia* sp.	CBS 100547*	KC343215	KC343941	KC344183	KC343457	KC343699
*D.tectonae*	Thailand	*Tectonagrandis*	MFLUCC 12-0777*	KU712430	KU749359	KU743977	KU749345	NA
*D.tectonendophytica*	Thailand	*Tectonagrandis*	MFLUCC 13-0471*	KU712439	KU749367	KU743986	KU749354	NA
*D.tectonigena*	China	*Tectonagrandis*	MFLUCC 12-0767*	KU712429	KU749371	KU743976	KU749358	NA
*D.tectonigena*	China	*Camelliasinensis*	LC6512	KX986782	KX999174	KX999214	KX999284	KX999254
*D.terebinthifolii*	Brazil	*Schinusterebinthifolius*	CBS 133180*	KC343216	KC343942	KC344184	KC343458	KC343700
*D.thunbergiicola*	Thailand	*Thunbergialaurifolia*	MFLUCC 12-0033*	KP715097	KP715098	NA	NA	NA
*D.tulliensis*	Australia	*Theobromacacao*	BRIP 62248a*	KR936130	KR936133	KR936132	NA	NA
*D.ueckeri*	USA	*Cucumismelo*	FAU656*	KJ590726	KJ590747	KJ610881	KJ612122	KJ659215
*D.unshiuensis*	China	*Fortunellamargarita*	CGMCC 3.17566*	KJ490584	KJ490463	KJ490405	NA	KJ490526
*D.unshiuensis*	China	*Caryaillinoensis*	CFCC 52594	MH121529	MH121571	MH121606	MH121447	MH121487
*D.unshiuensis*	China	*Caryaillinoensis*	CFCC 52595	MH121530	MH121572	MH121607	MH121448	MH121488
*D.vawdreyi*	Australia	*Psidiumguajava*	BRIP 57887a	KR936126	KR936129	KR936128	NA	NA
*D.vexans*	USA	*Solanummelongena*	CBS 127.14	KC343229	KC343955	KC344197	KC343471	KC343713
*D.viniferae*	China	*Vitisvinifera*	JZB320071*	MK341550	MK500107	MK500112	MK500119	NA
*D.vochysiae*	Brazil	*Vochysiadivergens*	LGMF1583*	MG976391	MK007526	MK007527	MK007528	MK033323
*D.xishuangbanica*	China	*Camelliasinensis*	CGMCC 3.18283*	KX986784	KX999176	KX999217	NA	NA
*D.xishuangbanica*	China	*Camelliasinensis*	LC6707	KX986783	KX999175	KX999216	NA	KX999255

Notes: NA, not applicable. * ex-type strains.

### ﻿Phylogeny

For the phylogenetic analysis, sequences of reference *Diaporthe* species and related taxa were downloaded from NCBI GenBank based on recent publications on the genus *Diaporthe* (Norphanphoun et al. 2022) (Table 2). Downloaded sequences were aligned together with the sequences obtained in the present study using MAFFT version 7.526 (Katoh and Standley 2013) and manually corrected using Bioedit 7.0.9.0 (Hall 1999). The phylogenetic analyses of the combined gene regions were performed using Maximum Likelihood (ML) and Bayesian Inference (BI) methods. ML was conducted using PhyML v. 3.0 (Guindon et al. 2010), with 1000 bootstrap replicates while BI was performed using a Markov Chain Monte Carlo (MCMC) algorithm in MrBayes v. 3.0 (Ronquist and Huelsenbeck 2003). Two MCMC chains, started from random trees for 1,000,000 generations and trees, were sampled every 100^th^ generation, resulting in a total of 10,000 trees. The first 25% of trees were discarded as burn-in of each analysis. Branches with significant Bayesian Posterior Probabilities (BPP) were estimated in the remaining 7500 trees. Phylogenetic trees were visualized with FigTree v.1.3.1 (Rambaut and Drummond 2010) and processed by Adobe Illustrator CS5. The nucleotide sequence data of the new taxa were deposited in GenBank (Table 2)

## ﻿Results

### ﻿Phylogenetic analyses

In the present study, we inferred a genus tree of *Diaporthe* covering a large proportion of sequence data available as last summarized by Norphanphoun et al. (2022). Two strains CFCC 58154 and GZ13B formed a clade in the *D.arecae* species complex, and the other strains CFCC 58151 and GZ13E in the *D.sojae* species complex.

**Figure 1.**
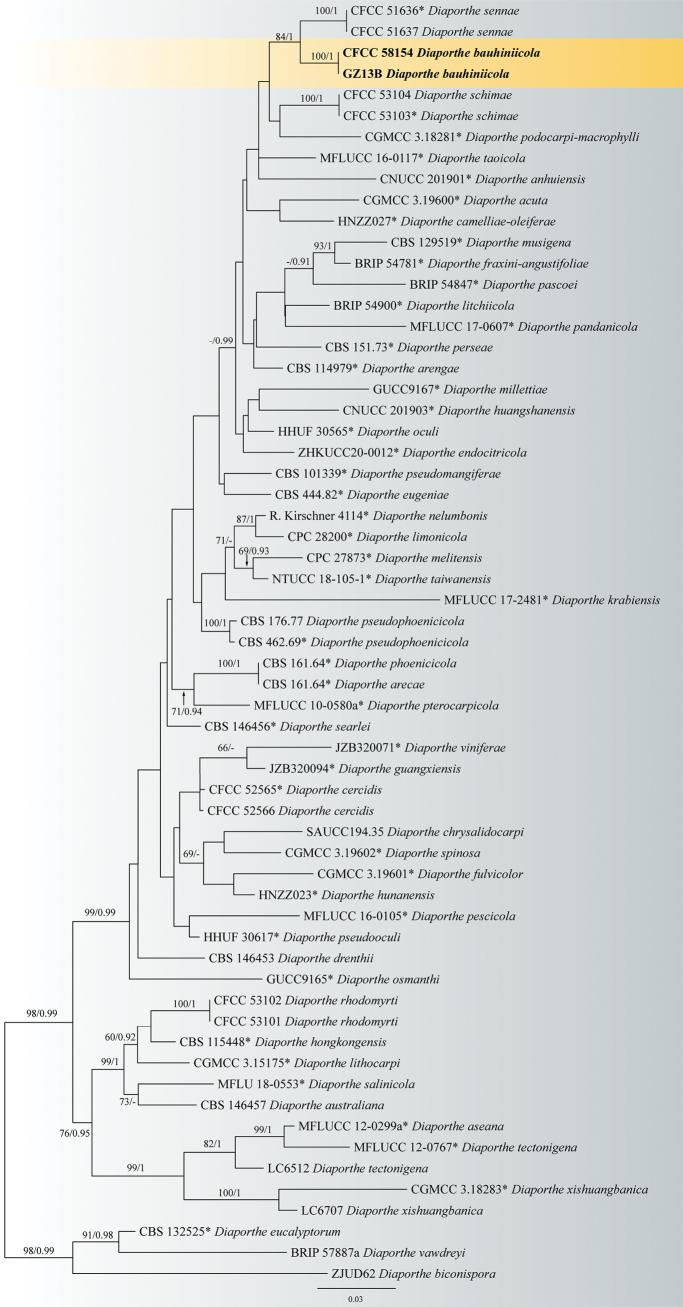
Phylogram of *Diaporthearecae* species complex resulting from a maximum likelihood analysis based on a combined matrix of ITS, *cal*, *his3*, *tef1* and *tub2* loci. Numbers above the branches indicate ML bootstrap values (left, ML BS ≥ 50%) and Bayesian posterior probabilities (right, BPP ≥ 0.9). Isolates from the present study are in bold and ex-type strains are marked with *.

**Figure 2.**
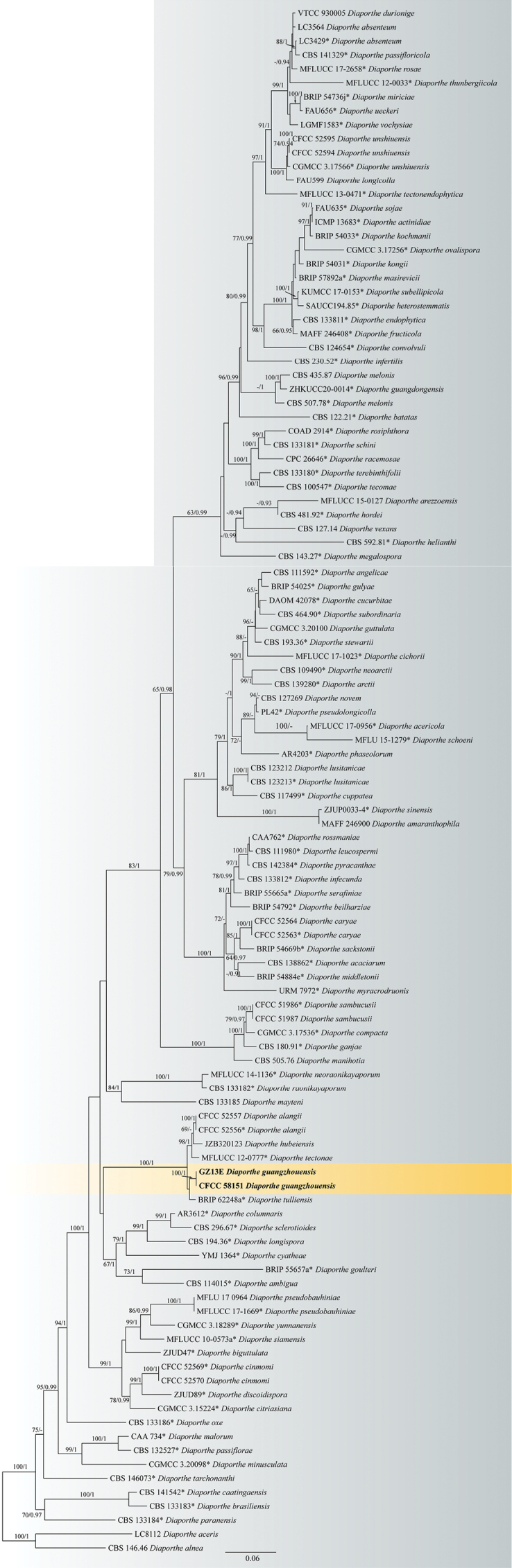
Phylogram of *Diaporthesojae* species complex resulting from a maximum likelihood analysis based on a combined matrix of ITS, *cal*, *his3*, *tef1* and *tub2* loci. Numbers above the branches indicate ML bootstrap values (left, ML BS ≥ 50%) and Bayesian posterior probabilities (right, BPP ≥ 0.9). Isolates from the present study are in bold and ex-type strains are marked with *.

In the *D.arecae* species complex, the combined sequence alignments comprised 61 strains, with *D.eucalyptorum* (CBS 13252), *D.biconispora* (ZJUD62) and *D.vawdreyi* (BRIP 57887a) as the outgroup taxa. The dataset comprised 2662 characters including alignment gaps (590 for ITS, 499 for *cal*, 485 for *his3*, 375 for *tef1* and 713 for *tub2*). CFCC 58154 and GZ13B from *Bauhiniavariegata* formed a distinct clade close to *D.sennae* (Fig. 1). In the *D.sojae* species complex, the combined sequence alignments comprised 166 strains (Fig. 2), with *D.aceris* (LC8112) and *D.alnea* (CBS 146.46) as the outgroup taxa. The dataset comprised 3025 characters including alignment gaps (602 for ITS, 592 for *cal*, 521 for *his3*, 483 for *tef1* and 827 for *tub2*). CFCC 58151 and GZ13E from *B.variegata* clustered in a distinct clade close to *D.tulliensis* (Fig. 2).

### ﻿Taxonomy

#### ﻿ Diaporthe bauhiniicola

Taxon classificationFungiDiaporthalesDiaporthaceae

Ning Jiang & Y.Q. Zhu sp. nov.

2D6AD0F1-0758-54ED-B0EB-B5BDDB93E656

 854183

Fig. 3

##### Holotype.

China • Guangdong Province, Guangzhou City, Luhu Park, 23°9'11.15"N, 113°16'46.01"E, 92 m asl, on diseased leaves of *Bauhiniavariegata*, 8 Aug 2022, Yong Li, Chengbin Wang & Yaquan Zhu, (holotype: CAF800094; ex-type culture: CFCC 58154).

**Figure 3.**
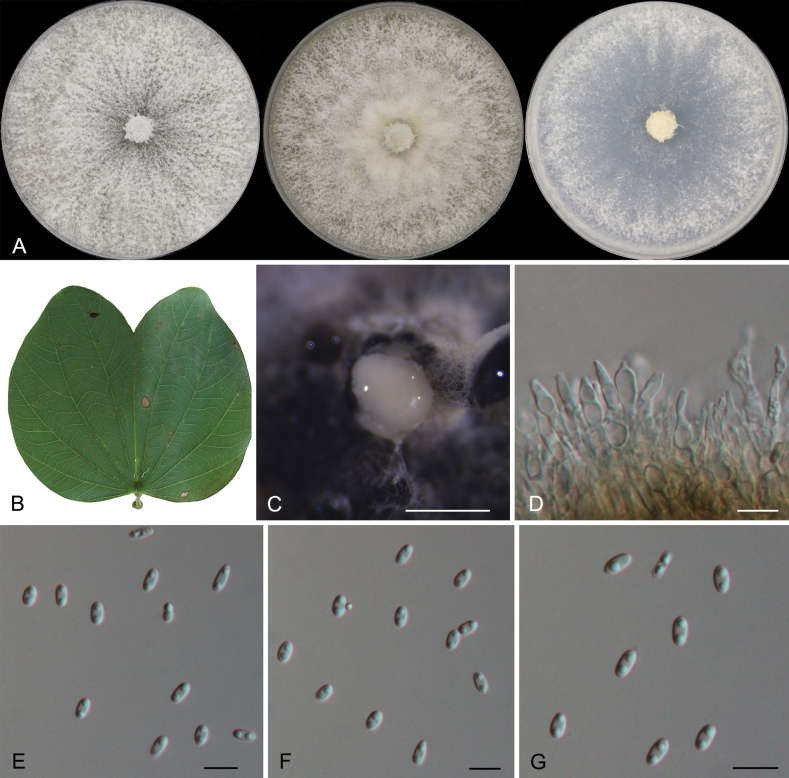
Morphology of *Diaporthebauhiniicola***A** colonies on PDA, MEA and SNA at 25 °C after 2 weeks **B** a diseased leaf of *Bauhiniavariegata***C** conidioma formed on PDA after 30 days **D** conidiogenous cells with attached alpha conidia **E–G** alpha conidia. Scale bars: 200 µm (**C**); 10 µm (**D–J**).

##### Etymology.

Named after the host genus, *Bauhinia*.

##### Description.

***Conidiomata*** formed on PDA pycnidial, scattered to aggregated, black, erumpent, raising above surface of culture medium, subglobose, 150–300 μm diam., exuding white or yellowish creamy conidial droplets from central ostioles after 30 days at 25 °C. ***Conidiophores*** reduced to conidiogenous cells. ***Conidiogenous cells*** hyaline, unbranched, septate, straight, slightly tapering towards the apex, 6.0–15.0 × 1.5–4.0 μm. ***Alpha conidia*** hyaline, aseptate, ellipsoidal to spindle-shaped, biguttulate or with one guttulate, 4.5–7.0 × 2.0–3.0 μm. ***Beta conidia and gamma conidia*** not observed. *Teleomorph* not observed.

##### Culture characteristics.

Colonies covering entire plate after 2 weeks. On PDA with profuse aerial mycelium, white surface, reverse fulvous. On MEA with fluffy aerial mycelium, dirty white surface, reverse ochreous. On SNA white sparse aerial mycelium, surface and reverse white.

##### Additional material examined.

China • Guangdong Province, Guangzhou City, Luhu Park, 23°9'11.15"N, 113°16'46.01"E, 92 m asl, on diseased leaves of *Bauhiniavariegata*, 8 Aug 2022, Yong Li, Chengbin Wang & Yaquan Zhu, living culture GZ13B.

##### Notes.

Two strains representing *Diaporthebauhiniicola* clustered in a clade distinct from its closest phylogenetic neighbour, *D.sennae* (Fig. 1). *D.sennae* has been reported from the host *Sennabicapsularis* in China (Yang et al. 2017). *D.bauhiniicola* differs from *D.sennae* by wider alpha conidia (4.5–7.0 × 2.0–3.0 μm in *D.bauhiniicola* vs. 5.0–6.5 × 1.5–1.8 μm in *D.sennae*) (Yang et al. 2017). *Diaporthebauhiniicola* differs from *D.sennae* in nucleotide sequence data (18/529 in ITS, 5/490 in *cal*, 15/351 in *tef1*, 14/677 in *tub2*) (Yang et al. 2017).

#### ﻿ Diaporthe guangzhouensis

Taxon classificationFungiDiaporthalesDiaporthaceae

Ning Jiang & Y.Q. Zhu sp. nov.

2A0959D8-088A-5FA5-A7A2-765C956730CF

 854184

Fig. 4

##### Etymology.

Named after the collection site of the type specimen, Guangzhou City.

##### Holotype.

China • Guangdong Province, Guangzhou City, Longdong straight street, 23°11'41.02"N, 113°22'8.33"E, 46 m asl, on diseased leaves of *Bauhiniavariegata*, 8 Aug 2022, Yong Li, Chengbin Wang & Yaquan Zhu, (holotype: CAF800095; ex-type culture: CFCC 58151).

**Figure 4.**
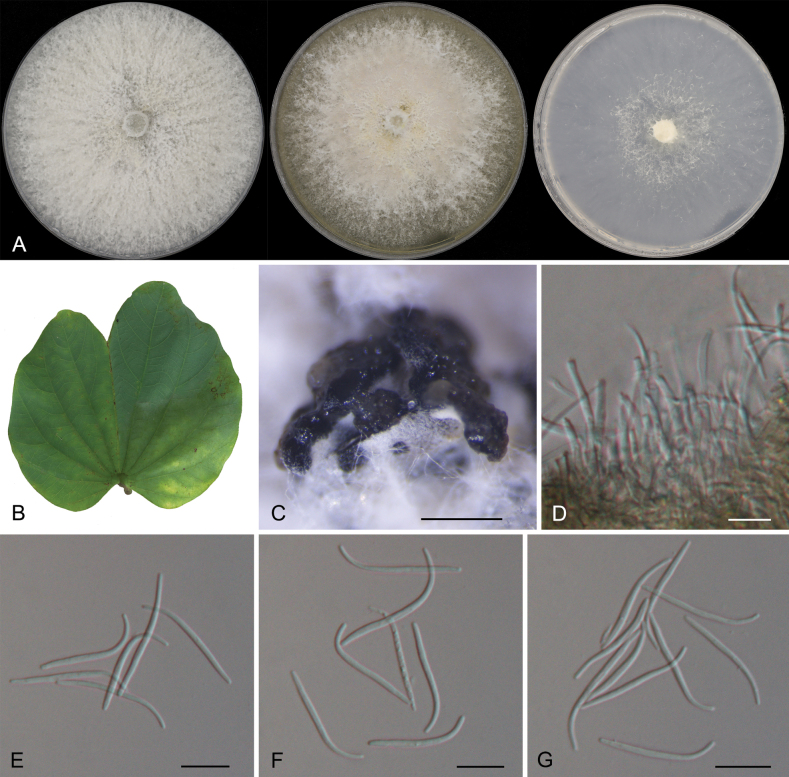
Morphology of *Diaportheguangzhouensis***A** colonies on PDA, MEA and SNA at 25 °C after 2 weeks **B** the leaf of *Bauhiniavariegata***C** conidiomata **D** conidiogenous cells with attached beta conidia **E–G** beta conidia. Scale bars: 200 µm (**C**); 10 µm (**D–J**).

##### Description.

***Conidiomata*** pycnidial, scattered to aggregated, black, erumpent, raising above surface of culture medium, subglobose, 150–450 µm diam, exuding white or yellowish creamy conidial droplets from central ostioles after 30 days at 25 °C. ***Conidiophores*** 12.5–24.5 × 1–2.5 μm, cylindrical, hyaline, unbranched, straight to sinuous. ***Conidiogenous cells*** densely aggregated, phiailidic, unbranched, straight or slightly curved, 5.5–10 × 2.0–7.5 μm. ***Beta conidia*** filiform, hyaline, straight or slightly curved, aseptate, 17.0–29.5 × 1.0–2.0 μm. ***Alpha conidia and gamma conidia*** not observed. ***Teleomorph*** not observed.

##### Culture characteristics.

Colonies covering entire plate after 2 weeks. On PDA with profuse aerial mycelium, white surface, reverse amber. On MEA with fluffy aerial mycelium, dirty white surface, reverse ochreous. On SNA white sparse aerial mycelium, surface and reverse white.

##### Additional material examined.

China • Guangdong Province, Guangzhou City, Longdong straight street, 23°11'41.02"N, 113°22'8.33"E, 46 m asl, on diseased leaves of *Bauhiniavariegata*, 8 Aug 2022, Yong Li, Chengbin Wang & Yaquan Zhu, living culture GZ13E.

##### Notes.

*Diaportheguangzhouensis* from the present study is phylogenetically close to *D.tulliensis* (Fig. 2). *Diaportheguangzhouensis* differs from *D.tulliensis* in nucleotide sequence data (5/526 in ITS, 9/347 in *tef1*, 13/711 in *tub2*) (Crous et al. 2015). In addition, host and distribution data are vital for species identification (*D.guangzhouensis* inhabiting *Bauhiniavariegata* in China vs. *D.tulliensis* inhabiting *Theobromacacao* in Australia) (Crous et al. 2015).

## ﻿Discussion

In the current study, phylogenetic analyses based on five combined loci (ITS, *cal*, *his3*, *tef1* and *tub2*), as well as morphological characters of the anamorph obtained in culture, revealed *D.bauhiniicola* and *D.guangzhouensis* spp. nov. from *Bauhiniavariegata*, which contributed to our knowledge of the diversity of *Diaporthe* species in China.

*Diaporthepseudobauhiniae* (syn. *D.chiangraiensis*, *Chiangraiomycesbauhiniae*) was described as a saprobic fungus on branches of *Bauhinia* sp. in Thailand (Senanayake et al. 2017). *D.bauhiniae* was introduced from branches of *B.purpurea* in China (Yang et al. 2021). Hence, a total of four species of *Diaporthe* have been recorded from the host genus *Bauhinia*. Phylogenetically, *D.bauhiniae* belongs to *D.varians* species complex; *D.bauhiniicola* belongs to *D.arecae* species complex; *D.guangzhouensis* and *D.pseudobauhiniae* belong to *D.sojae* species complex (Figs 1, 2) (Norphanphoun et al. 2022). Furthermore, *D.guangzhouensis* and *D.pseudobauhiniae* formed different clades in *D.sojae* species complex (Fig. 2). Morphologically, *D.bauhiniicola* has larger alpha conidia than *D.pseudobauhiniae*, but longer alpha conidia than *D.bauhiniae* (4.5–7.0 × 2.0–3.0 μm in *D.bauhiniicola* vs. 3–5 × 2–4 μm in *D.pseudobauhiniae* vs. 7.5–14 × 1.5–3 μm in *D.bauhiniae*) (Senanayake et al. 2017; Yang et al. 2021). *D.guangzhouensis* shares similar beta conidia size with *D.pseudobauhiniae* that are shorter and wider than *D.bauhiniae* (17.0–29.5 × 1.0–2.0 μm in *D.guangzhouensis* vs. 18–38 × 1.5–2 μm in *D.pseudobauhiniae* vs. 25–43 × 1 µm in *D.bauhiniae*) (Senanayake et al. 2017; Yang et al. 2021). Another species named *Phomopsisbauhiniae* was recorded on the branches of *Bauhiniavariegata* in Spain, however, this species was only studied in morphology and has not been combined in *Diaporthe* (Uecker 1988). *Diaporthebauhiniicola* has shorter but wider alpha conidia than *P.bauhiniae* morphologically (Uecker 1988). The molecular analyses are necessary for the species *P.bauhiniae* based on the ex-type culture in the future.

The initial species concept of *Diaporthe* based on the assumption of host-specificity, resulted in the introduction of more than 1000 taxa (http://www.indexfungorum.org/). However, more than one species of *Diaporthe* have been often discovered from the same host (Gomes et al. 2013; Guarnaccia and Crous 2017; Guarnaccia et al. 2020; Guo et al. 2020). For example, *D.caryae* and an additional 18 *Diaporthe* species are associated with pear shoot canker in China (Guo et al. 2020); *D.sennae* and *D.sennicola* inhabit branches of *Sennabicapsularis* causing canker diseases (Yang et al. 2017). The current study further supports this phenomenon.

*Diaporthe* is considered as a species-rich genus. Nevertheless, an emerging perspective posits that the quantity of recognized *Diaporthe* species may have been substantially overestimated. The *D.amygdali* species complex has been proven a single species evidenced from the genealogical concordance phylogenetic species recognition principle (GCPSR) and coalescence-based models: general mixed yule-coalescent (GMYC) and poisson tree processes (PTP), with several species becoming synonyms (Hilário et al. 2021b). Similarly, several species in the *D.eres* species complex such as *D.betulae* and *D.padina* were treated as synonyms (Hilário et al. 2021a). A comprehensive study is necessary to clarify species boundaries of *Diaporthe* in the future. This will help improve our understanding of the species concept within this genus.

## Supplementary Material

XML Treatment for Diaporthe bauhiniicola

XML Treatment for Diaporthe guangzhouensis

## ﻿Additional information

### Author ORCIDs

Yaquan Zhu https://orcid.org/0000-0002-3296-239X

Han Xue https://orcid.org/0000-0003-0414-6237

Yong Li https://orcid.org/0000-0002-4406-1329

Ning Jiang https://orcid.org/0000-0002-9656-8500

### Data availability

All of the data that support the findings of this study are available in the main text.
